# Adaptive Behavior in Young Children with Neurofibromatosis Type 1

**DOI:** 10.1155/2013/690432

**Published:** 2013-11-19

**Authors:** Bonita P. Klein-Tasman, Alina M. Colon, Natalie Brei, Faye van der Fluit, Christina L. Casnar, Kelly M. Janke, Donald Basel, Dawn H. Siegel, Jasmine A. Walker

**Affiliations:** ^1^University of Wisconsin - Milwaukee, Milwaukee, WI 53201, USA; ^2^Medical College of Wisconsin, Milwaukee, WI 53226, USA

## Abstract

Neurofibromatosis-1 is the most common single gene disorder affecting 1 in 3000. In children, it is associated not only with physical features but also with attention and learning problems. Research has identified a downward shift in intellectual functioning as well, but to date, there are no published studies about the everyday adaptive behavior of children with NF1. In this study, parental reports of adaptive behavior of 61 children with NF1 ages 3 through 8 were compared to an unaffected contrast group (*n* = 55) that comprised siblings and community members. Significant group differences in adaptive skills were evident and were largely related to group differences in intellectual functioning. In a subsample of children with average-range intellectual functioning, group differences in parent-reported motor skills were apparent even after controlling statistically for group differences in intellectual functioning. The implications of the findings for the care of children with NF1 are discussed.

## 1. Introduction

Neurofibromatosis type 1 (NF1) is an autosomal dominant genetic disorder occurring in approximately 1 in 3000 live births. Mutation of the NF1 gene (chromosome 17q11.2), neurofibromin 1, results in activation of the RAS signaling pathway which has far-reaching effects in many cellular and neurodevelopmental processes [1]. As a tumor suppressor, reduced production of neurofibromin results in the characteristic tumors that define NF1. Common physical characteristics include café au lait spots, cutaneous or subcutaneous neurofibromas, axiliary freckling, and Lisch nodules, with diagnosis made based on physical characteristics [2]. About half of cases are inherited from a parent and about half are sporadic mutations. The disorder occurs equally in both males and females and in all ethnic groups. While physical complications exist in NF1, there are also psychological complications. Children with NF1 are at increased risk for cognitive, attention, and learning problems [3–6]. Features identified in individuals with NF1 include poor performance in the areas of Language Expression and comprehension, written language, reading accuracy, mathematics, and fine motor skills [7–10]. However, there has been little comprehensive examination of the functional impact of these difficulties in everyday life.

The goal of this study is to evaluate the pattern of adaptive functioning in young children with NF1 as an indication of the functional impact of the difficulties seen in NF1. The American Association on Mental Retardation (AAMR) defines adaptive behavior as “the collection of conceptual, social, and practical skills that have been learned by people in order to function in their everyday lives (p. 73)” [11]. Sattler and Hoge [12] further elaborate, indicating that conceptual skills are seen in receptive as well as expressive language, reading, writing, and handling money, that social skills involve establishing friendships, social reasoning, interpretation, and comprehension, and examples of practical skills include actions such as bathing, preparing food, washing dishes, basic housekeeping, and taking medicine. Hence, adaptive behavior relates to practical skills and abilities needed to function independently in home, social, school, work, and community settings [13]. Most measures of adaptive behavior in the preschool and early school-age years include measures of social, communication, daily living, and community living skills and also include motor skills especially in young children, most frequently as described by parents. Studies of relations between adaptive behavior and cognitive functioning suggest that these are closely coupled [14]. 

There have been few investigations of broad adaptive behavior in children with NF1. As part of a much larger descriptive study, Dilts and colleagues [15] found that children with NF1 ages 6 through 17 (*n* = 20) were less functionally independent than were unaffected siblings (*n* = 20) in the areas of broad independence, motor skills, social and communication skills, and community living skills, but not in personal living skills, with the largest group difference in motor skill development. Using the adaptive scales of the Achenbach Teacher Report Form [16], with 79 children ages 8 through 16, Barton and North [17] found that children with NF1 had significantly worse adaptive functioning than children without NF1, as well as poorer adaptive functioning when compared to normative data. The adaptive scales of the Achenbach use an open-ended format to ask parents to list activities, hobbies, and chores and are not considered an adequate comprehensive measure of adaptive functioning [12, 18], rather serving as a screening tool. In sum, the study of adaptive behavior in NF1 is sparse, and there have been no studies of adaptive behavior in younger children.

There is also an accumulation of prior research suggestive of difficulties with the separate conceptual, social, and motor skills components of adaptive behavior in children with NF1, using structured standardized assessments. Related to conceptual skills, a downward shift in intellectual functioning and increased risk of learning difficulties is consistently described based on direct measurement of these abilities with standardized assessment methods. The vast majority of children with NF1 show overall cognitive functioning in the average range, although specific areas of difficulty are generally noted for close to half of people with NF1 [19, 20]. Language and communication difficulties are also commonly described [17], with some beginning evidence that these difficulties are discernible on standardized individually administered language assessments even in the preschool years [21–23]. Related to social skills, a number of studies have demonstrated social skills difficulties, based on parent and teacher report as well as peer ratings [15, 17, 24, 25] in comparison to same-aged peers or normative data, even for children as young as kindergarteners [17]. In the area of motor skills, as mentioned, Dilts and colleagues [15] pointed specifically to the prominence of everyday difficulties in motor skills. Several other investigations have similarly revealed that motor difficulties are common for children with NF1 based on parental report [26] and lab-based measures [27–30], although these difficulties typically do not reach the level of clear impairment. 

Hence, difficulties with many of the components and contributors to overall adaptive functioning have been described in the literature about school-aged children with NF1. However, few have been overarching studies of all of these domains together, focusing on the day-to-day functional impact of these difficulties from parents' perspectives. Moreover, the literature about adaptive functioning in young children with NF1 is sparse; there have been no published studies of adaptive behavior in young children with NF1 using one of the commonly used comprehensive measures of adaptive behavior. In the current study, adaptive functioning of children 3 through 8 years is examined in comparison to unaffected children using a broad parent interview measure covering social, communication, daily living, community, and motor skills domains. Given that a downward shift in intellectual functioning is seen in NF1 and adaptive functioning is closely coupled with intellectual functioning, it is hypothesized that the children with NF1 will show weaker adaptive skills in comparison to same-aged peers. In other words, it is expected that the cognitive difficulties in children with NF1 will translate into difficulties in everyday functioning. Based on prior research, it is expected that motor skills and social/communication skills will be particularly affected. Group differences in adaptive behavior are expected to be largely driven by intellectual functioning.

## 2. Method

### 2.1. Participants

Participants were 61 children with NF1 (36 male, 25 female; 24 familial cases, 37 sporadic) and 55 unaffected children (34 male, 21 female) consisting of 30 siblings of children with NF1 and 25 community participants between the ages of 3 and 8 years. All children with NF1 and siblings were included in the sample regardless of intellectual functioning. Among the unaffected children recruited from the community (*n* = 36), there was an overrepresentation of children with above-average intellectual functioning, so those with a General Cognitive Ability score over 115 were excluded from the study (*n* = 11). The sibling and community participants were compared on a number of relevant variables prior to combining them into one group. The groups did not differ in age, cognitive functioning, gender distribution, or adaptive behavior at the overall or domain level. Therefore, the two groups were combined to maximize power rather than using a three-group analytic approach. Additional participant characteristics are described in Table 1.

Participants with NF1 and their siblings were recruited from neurofibromatosis specialty clinics in the Midwest designed for the medical management and anticipatory guidance regarding NF1. Participants were approached by study personnel at their yearly medical check-in; participants attending clinic for initial diagnostic assessment were not approached. It was emphasized to families that the purpose of the study was to describe cognitive and psychosocial functioning of young children with NF1 and that it was important that participants with a range of functioning be included—both children experiencing developmental difficulties and children for whom no difficulties were currently noted. Hence, the assessments conducted were not conducted due to clinical concerns about neurodevelopmental functioning. The response rate was high; only two of the families approached declined to participate. 

Inclusion criteria included the following: (1) clinical diagnosis of NF1; (2) primary language English. A range of severity of NF1 was represented. Three participants had identified optic glioma for which chemotherapy treatment was provided together with shunting for hydrocephalus, one had hydrocephalus alone, and four had asymptomatic optic glioma that were untreated. A physician completed severity report, based on a medical severity measure [31], was available for 50 of the 61 participants indicating minimal severity for the vast majority of the participants (*n* = 30), mild severity for 11 participants, moderate severity for 8 participants, and high severity for 1 participant. Analyses were conducted both including and excluding those with a history of optic glioma and/or hydrocephalus, with no substantial differences in findings.

Community participants were recruited by posting fliers in areas frequented by families with young children (coffee shops, YMCA, and libraries). Participants were ineligible for participation if they had an identified neurodevelopmental disorder or received special education services at school. 

### 2.2. Measures

*The Scales of Independent Behavior Revised* (SIB-R; [13]) is a widely used comprehensive measure of adaptive behavior. It measures functional independence in school, home, work, and community environments. The assessment is arranged into 4 clusters which in turn are comprised of multiple subscales (Table 4 contains a listing of the domains and subdomains). The Broad Independence (BI) index of the measure provides an overall assessment of adaptive behavior, the domain scores provide a sense of functioning in specific areas, and subdomain scores provide a sharper view of specific abilities. Standard scores (*M* = 100, SD = 15) are available at the domain level, and qualitative descriptors are available at the subdomain level. The SIB-R was normed on approximately 1,700 individuals who range in age from infants less than three months old to mature adults. Parents were interviewed and asked to rate their children's behavior using a four point rating scale which ranges from *0 (Never or Rarely (does)*—*even if asked)* to *3 (Does Very Well*—*always or almost always*—*without being asked). *In this study, standard scores in Broad Independence and at the domain level were examined, and qualitative descriptors were examined at the domain and subdomain levels. The qualitative descriptors indicate how difficult age-level tasks in the given domain are expected to be for the child, based on a Relative Mastery Index. The number of children for whom age-level tasks are expected to be difficult was examined. 

*The Differential Ability Scales—Second Edition* (DAS-II; [32]) is a comprehensive individually administered assessment of cognitive abilities that are important to learning including verbal reasoning, nonverbal reasoning, and spatial abilities. It yields a General Cognitive Ability (GCA) standard score, which reflects overall conceptual/reasoning ability, and is similar to an Intelligence Quotient. This measure is a validated measure of cognitive functioning for use with children ages 2 years, 6 months through 17 years, 11 months [33]. This study used the Early Years Form for children three to eight years of age. 

### 2.3. Procedure

The SIB-R was individually administered to parents as a structured face-to-face interview, as standardized. Children were individually administered the DAS-II, as the first part of a larger battery that included age-appropriate lab-based experimental measures of temperament and emerging executive functioning, with specific measures appropriate to chronological age. Parents also completed additional questionnaires assessing psychosocial functioning that are not included here as the versions of the measures varied across the age range of the current study. This work was conducted according to an IRB-approved protocol.

## 3. Results

The data were analyzed using SPSS. Findings at *P* < .05 were generally considered significant. When multiple comparisons were conducted (i.e., more than 4), an adjusted alpha level of *P* < .01 was used. Skewness and kurtosis were acceptable for all variables for both groups of participants. For *t*-test, Levene's test for equality of variances occasionally indicated a violation of this assumption, but pooled variance *t*-test showed identical findings to traditional *t*-test. Statistical assumptions for all other analyses were met.

### 3.1. Individual Differences

#### 3.1.1. Age

Bivariate Pearson correlations between age and scaled scores on the SIB-R were examined (see Table 3). For the NF1 group, there were no significant correlations with age. For the unaffected participants, a significant correlation between age and Personal Living standard score was observed, with older children showing stronger Personal Living skills relative to their peers. 

#### 3.1.2. Gender

No group differences in gender were found at the domain level or scale level.

#### 3.1.3. IQ

*t*-test indicated that a significant group difference in GCA was observed (*t*[114] = 6.17, *P* < .001). Bivariate Pearson correlations with IQ were also examined (see Table 3). For both the NF1 group and the unaffected group, there were significant correlations between IQ and all adaptive behavior standard scores, with the exception of motor skills (*r*(61) = .318, *P* = .013 for the NF1 group, *r*(61) = .310, *P* = .021) for the unaffected participants. 

### 3.2. Group Differences in Adaptive Behavior

Based on *t*-tests, significant group differences were found in overall adaptive functioning, motor skills, and community living skills, with a trend towards a significant difference in social/communication (see Table 2). To examine whether group differences in adaptive functioning were largely driven by group differences in intellectual functioning, a MANCOVA controlling for GCA was conducted. This analysis showed no significant group differences in overall adaptive functioning or any specific domains once intellectual functioning was taken into account, although there was a trend for group differences in motor skills (*F*[1,116] = 3.30, *P* = .072). 

To further probe adaptive functioning problems related to NF1 and minimize the potential role of decrements in intellectual functioning through *experimental design* rather than *statistical control*, the MANOVA was rerun including only participants in both groups with intellectual functioning in the average range or stronger (GCA > 84). This subgroup was comprised of 45 children with NF1 (27 male, 18 female) and 54 unaffected children (33 male, 21 female). The two subgroups continued to show significant differences in GCA (*t*(97) = 4.48, *P* < .001) but did not differ on SES (*t*(93) = .36, *P* = .72) or age (*t*(97) = .94, *P* = .35). The children with NF1 exhibited significantly lower functioning in motor skills (*t*(97) = 2.73, *P* = .008).

The proportion of the children in each group showing difficulties in adaptive functioning was also examined and compared across the groups (see Table 4). Difficulties were identified based on Relative Mastery Index scores classified by the SIB-R as reflecting difficulty. At the domain level, group differences in motor functioning and in Broad Independence were noted. At the subdomain level, group differences were present for gross and fine motor skills, language expression, and time and punctuality.

## 4. Discussion

In this examination of everyday adaptive behavior of young children with NF1, children with NF1 showed weaker adaptive behavior than same-aged peers at the overall level and in motor and community living skills, suggesting that the well-documented general intellectual difficulties seen in children with NF1 translate into difficulties in real-world functioning. Analogous to findings regarding intellectual functioning in NF1, a slight downward shift in average adaptive functioning was observed. While children with NF1 do not show stark areas of impairment in adaptive functioning, these results nevertheless provide evidence that NF1 leads to a dampening of adaptive functioning and that, like in other populations, decrements in adaptive functioning appear to be closely intertwined with general intellectual difficulties. 

There is controversy regarding the appropriateness of controlling for intellectual functioning when examining the behavioral phenotype associated with neurodevelopmental disorders [34] and for NF1 specifically [35], with the argument that IQ is a global functioning variable and that controlling for this general outcome variable can lead to “meaningless” findings, given that IQ would be expected to be related to many other variables, including adaptive behavior. Therefore, some discussion of group differences in adaptive functioning, before considering patterns of findings taking into account the role of intellectual functioning, is warranted. First, group differences in adaptive behavior, across the majority of the domains, were noted. While mean scores indicated functioning in the average range for the NF1 group, examinations of the frequency of difficulties in each of the domains and subdomains revealed that more children with NF1 than children in the contrast group show adaptive difficulties in all areas. At the subdomain level, difficulties with both fine and gross motor skills and with language expression were most notable. Practitioners may find that administration of measures of adaptive behavior to parents is useful to identify children with NF1 with motor or language difficulties, who would benefit from more comprehensive assessment and developmental support.

In comparison to the only other study broadly examining adaptive functioning in children with NF1 [15], the level of adaptive functioning in this sample was considerably higher, especially in the motor skills domain. More specifically, mean adaptive behavior in the Dilts study was 88, while in the current study it was 96. At the domain level, the greatest difference in findings between the studies is in the motor domain; Dilts and colleagues found that mean motor functioning was in the borderline range, while in the current study it was in the average range. Findings in the other domains are largely analogous. While no age effects were seen in the current study, it remains possible that motor difficulties might be seen as becoming more pronounced over Time if a larger age span was considered. Alternatively, this difference may relate to differences in ascertainment approaches or to improvements in early identification of children with NF1 without significant symptomatology. It is possible that NF1 is now being diagnosed at younger ages by physicians and that children with physical signs of NF1 but no developmental difficulties may be more likely to be identified now than at the Time of Dilts and colleagues' study.

While there is indeed controversy about statistically controlling for intellectual functioning, as discussed above [34], inclusion of analysis statistically controlling for intellectual functioning is nonetheless informative as it demonstrates a potentially strong role for intellectual functioning in adaptive skills. These abilities are closely intertwined for children with NF1, as they are in the general population. It is also notable that motor functioning was not significantly correlated with intellectual functioning. Based on both the statistical and experimental design approaches to controlling for intellectual functioning, there is suggestive evidence that NF1 has an impact on everyday motor functioning that is separate from its impact on intellectual functioning. Motor functioning is indeed a very commonly noted area affected by NF1 [27, 28, 36]. Even for those children who have average or above-average cognitive functioning, there may still be additional difficulties with motor skills in comparison to same-aged peers. These motor difficulties may translate into greater frustration with school-based tasks such as learning handwriting and may also potentially affect recreational skills such as participation in group-based sports activities. Further research about the effects of motor functioning difficulties on the lives of children with NF1 is warranted.

## 5. Conclusions

In sum, this is one of the first studies to comprehensively examine adaptive behavior in young children with NF1 in comparison to same-aged peers. Mild decrements in adaptive behavior were observed across the range of adaptive behavior domains, with relative difficulty with motor skills remaining even after intellectual functioning was taken into account. Difficulties with motor skills and Language Expression were the most common everyday difficulties. One limitation of this study is the reliance on parental report alone to characterize adaptive behavior. Studies that include measurement of real-world behavior and examination of relations between parental report and lab-based measurement of the abilities examined are important next steps. Additionally, potential relations with other cognitive skills such as emerging executive functioning and attention should be explored. Finally, further research about the developmental trajectory of early motor difficulties in particular is also needed. 

## Figures and Tables

**Table 1:** Demographic data.

	NF1 (*n* = 61)	Unaffected (*n* = 55)
Intellectual functioning (GCA)	91.51 (SD = 13.41)	105.75 (SD = 11.12)
Age (Mean, SD)	4 years, 11 months (SD = 19.59 months)	5 years, 3 months (SD = 19.28 months)
Gender		
Male	36	34
Female	25	21
Ethnicity		
Caucasian	45	46
African-American	6	4
Latino	6	1
Asian	1	2
Mixed ethnicity	3	2
Maternal level of education		
High school	11	5
Higher education	50	50
Hollingshead SES index	33.26 (SD = 16.05)	36.29 (SD = 14.66)

**Table 2:** Descriptive statistics of adaptive skills by group.

Domain	NF1		Unaffected		*t* value	*P* value
	Mean	SD	Mean	SD		
Motor	96.57	17.46	108.05	13.38	−3.94	<.001**
Social/communication	99.89	16.90	104.84	12.35	−1.78	.077
Personal living	96.52	16.82	99.42	12.79	−1.03	.303
Community living	92.43	18.79	98.44	11.99	−2.03	.045
Broad independence	96.03	17.39	103.65	96.03	−2.74	.007*

*P < .01; **P < .001.

**Table 3:** Correlations between adaptive functioning standard scores and age and intellectual functioning by group.

Scale	Age				GCA			
	NF1		Unaffected		NF1		Unaffected	
	*r*	*P*	*r*	*P*	*r*	*P*	*r*	*P*
Motor	−.297	.020	.179	.191	.318	.013	.310	.021
Social/communication	.152	.243	.016	.909	.541	<.001**	.432	.001*
Personal living	.183	.157	.367	.006*	.432	.001*	.364	.006*
Community living	−.015	.911	−.006	.964	.437	<.001**	.401	.002*
Broad independence	.026	.841	.173	.207	.512	<.001**	.492	<.001**

*P < .01; **P < .001.

**Table 4:** Percent of participants with adaptive functioning characterized as in the difficult, limited, or very limited range.

Subdomain/DOMAIN	NF1 group	Unaffected group	*χ* ^2^ value	*P* value
Gross motor	26.23	1.82	13.78	<.001**
Fine motor	34.43	12.73	7.44	.006*
MOTOR	26.22	5.45	9.11	.003*

Social interaction	4.92	1.82	.84	.361
Language comprehension	16.39	3.64	5.08	.024
Language expression	49.18	14.55	15.75	<.001**
SOCIAL/COMMUNICATION	21.31	7.27	4.56	.033

Eating and meal preparation	31.15	16.36	3.45	.063
Toileting	37.70	16.36	6.59	.010
Dressing	44.26	29.09	2.85	.091
Personal self-care	26.23	14.55	2.41	.121
Domestic skills	6.56	9.09	.26	.611
PERSONAL LIVING	24.59	7.27	6.33	.012

Time and punctuality	37.70	14.55	7.92	.005*
Money and value	42.62	25.45	3.77	.052
Work skills	36.07	18.18	4.63	.030
Home/community orientation	54.10	56.36	.06	.806
COMMUNITY LIVING	39.34	20.00	5.95	.015

BROAD INDEPENDENCE	24.59	5.45	8.08	.004*

*P < .01; **P < .001.
